# Qualitative analysis of patient interviews on the burden of neuronopathic Gaucher disease in Japan

**DOI:** 10.1186/s13023-022-02429-z

**Published:** 2022-07-19

**Authors:** Yuta Koto, Aya Narita, Shinichi Noto, Midori Ono, Anna Lissa Hamada, Norio Sakai

**Affiliations:** 1 Child Healthcare and Genetic Science Laboratory, Division of Health Sciences, Osaka University Graduate School of Medicine, 1-7 Yamadaoka, Suita, Osaka 565-0871 Japan; 2 Division of Child Neurology, Institute of Neurological Science, Faculty of Medicine, Tottori University, 36-1 Nishi-cho, Yonago, Tottori 683-8504 Japan; 3 Department of Rehabilitation, Niigata University of Health and Welfare, 1398 Shimami-cho, Kita-ku, Niigata, Niigata 950-3198 Japan; 4 Japan Medical Office, Takeda Pharmaceutical Company Limited, 1-1 Nihonbashi-Honcho 2-chome Chuo-ku, Tokyo, 103-8668 Japan

**Keywords:** Gaucher disease, Interviews as topic, Patient reported outcome measures, Qualitative research, Burden of disease, Japanese, Neuronopathic Gaucher disease

## Abstract

**Background:**

Gaucher disease (GD) is a rare, autosomal recessive lysosomal storage disorder that adversely affects life expectancy and health-related quality of life (HRQOL). Although HRQOL questionnaires are available for type 1 GD, they are not suitable for patients with the neuronopathic types 2 and 3 GD who have neurological symptoms that develop during early childhood or adolescence. Here we report the development of a language-validated HRQOL questionnaire specifically for patients with neuronopathic types 2 and 3 GD in Japan, which is the first step toward HRQOL questionnaire provision for all types of GD in the future.

**Methods:**

In February and March 2021, semi-structured interviews were conducted by the authors (supported by qualified interviewers) with patients and/or their caregivers (for patients < 16 years old) who were recruited from a Japanese patient association, the Association of Gaucher Disease Patients in Japan. Qualitative analysis of interview transcripts was used to identify major themes and key topics within those themes. Hierarchical cluster analysis and co-occurrence network analysis were performed to map relationships between commonly occurring words. The study is registered at the UMIN Clinical Trials Registry (https://www.umin.ac.jp/ctr/index.htm [UMIN000042872]).

**Results:**

Three main themes emerged from qualitative analysis: treatment status, patient burden, and social support systems. Key topics within each theme included hearing impairment, visual impairment, difficulty swallowing, difficulty speaking, involuntary movement of extremities, epileptic seizures, and body aches (treatment status); anxiety about symptoms, difficulty with exercise and work, anxiety about continuing treatment, anxiety about going out, and tiredness from hospital visit or treatment (patient burden); and dissatisfaction about government service, lack of social support, and information exchange in the patient association (social support systems). Commonly used words and the relationships between words identified through the hierarchical cluster and co-occurrence network analyses supported these themes and topics.

**Conclusions:**

The themes and topics identified in this analysis were specific to patients with types 2 and 3 GD and will be used to inform the development of a HRQOL questionnaire specifically for patients with all GD types.

**Supplementary Information:**

The online version contains supplementary material available at 10.1186/s13023-022-02429-z.

## Background

Gaucher disease (GD) is a rare, inherited, autosomal recessive lysosomal storage disorder with a worldwide prevalence of 0.7 to 1.75 per 100,000 [1]. Three major subtypes of GD have been described based on the presence or absence of neurological symptoms [2, 3]. Type 1 GD, called non-neuronopathic GD, is the most common in non-Japanese populations, accounting for approximately 94% of cases [4]. Types 2 and 3 GD, collectively known as neuronopathic GD (nGD), are less common. The main neurological symptoms of nGD include seizure, abnormal eye movements, and developmental delay, in addition to enlargement of the liver/spleen. Type 2 GD progresses rapidly, and affected infants have a very short life (2–3 years) despite the best supportive care [1, 5]. Type 3 GD is associated with a later onset in childhood than is seen in type 2. In Japan, a total of 211 patients are estimated to have GD [6]. However, in contrast to other countries, the proportion of patients with nGD in Japan is higher, accounting for approximately 60% of cases [6–8].

Management goals for patients with GD should address treatment of the various clinical manifestations, improvement of patients’ quality of life (QOL), and early detection of long-term complications and comorbidities [5]. Assessment of the effectiveness of treatment requires a holistic approach to data collection that includes the perspectives of both patients and their caregivers. Despite the importance of QOL in the management of patients with GD, limited information is available on the burden of nGD in patients and caregivers, as qualitative studies conducted to date have almost exclusively included patients with type 1 GD [9–11]. Although GD-specific patient-reported outcome measures (PROMs) have been developed, these questionnaires are focused on patients with type 1 GD [12–14] and are not suitable for capturing symptoms and comorbidities of patients with nGD. In addition, although a caregiver-reported outcome measure is currently under development for other lysosomal diseases, there is no such tool for caregivers of patients with GD [15]. In order to gain a more comprehensive understanding of the burden of GD, disease-specific outcome measures should include patient-reported items specific to nGD and caregiver-specific questions. The availability of nGD-specific questionnaires would be of benefit not only in countries with a higher prevalence of nGD, such as Japan, but also in countries with a limited number of patients with nGD.

The main purpose of this research is to develop a new, language-validated, health-related QOL (HRQOL) questionnaire for patients with GD and their caregivers, to evaluate the burden of GD, including types 2 and 3 neurological symptoms, which have not been covered to date. For content validity of the HRQOL questionnaire, qualitative research aimed at interpreting the experience of patients and their caregivers is essential [16]. Here we report the first stage in the development of the HRQOL questionnaire, which comprised qualitative analysis of interviews with patients with nGD and/or their caregivers, followed by hierarchical cluster analysis and co-occurrence network analysis, in order to identify keywords, major themes, and specific topics related to disease burden.

## Results

### Patient demographics

Eight patients (six male, two female) recruited from a patient association participated in the interview: four with type 2 GD (four caregivers) and four with type 3 GD (two patients and two caregivers) (Table 1). All four patients with type 2 GD were 2‒11 years old, whereas two patients with type 3 GD were adults and two were < 10 years old. All patients were diagnosed with GD at between 1 month and 3 years of age, except one who was diagnosed with type 3 GD at 18 years of age. Six patients started treatment relatively quickly after diagnosis at between 1 month and 3 years of age, but treatment was delayed in the other two patients because of a lack of appropriate treatment at the time. Three patients were bedridden, including one patient with type 2 GD who had tracheostomy and gastrostomy, three patients were able to walk without assistance, one patient needed nursing assistance, and one patient had no information about activities of daily living. The caregivers were the patients’ fathers or mothers. Each 1:1 interview lasted 30–40 min.

**Table 1:** Summary of interviewee characteristics

Characteristic	N = 8
Age range, years	2–57
Children (< 18 years)	6
Adults (≥ 18 years)	2
Age at diagnosis	
1 month–3 years	7
18 years	1
Sex	
Male	5
Female	3
Gaucher disease type	
Type 2	4
Type 3	4
Daily activities	
Bedridden	3
Walk without assistance	3
Nursing assistance	1
No information	1
Interviewee	
Patient	2
Caregiver	6

Data are number, except where indicated

### Themes

Three themes that were proposed in the interview guide and confirmed by the interview transcripts as the main categories of disease burden were analyzed further: “treatment status”, “patient burden﻿” (including physical, psychological, or economic burden caused by disease), and “social support systems”.

#### Treatment status

Qualitative analysis of the verbatim transcripts revealed seven main topics related to treatment status that were commonly mentioned by patients and/or caregivers (Table 2). These topics focused on the impact of physical symptoms of GD on daily life rather than the treatments themselves and included hearing impairment, visual impairment, difficulty swallowing, difficulty speaking, involuntary movement of extremities, epileptic seizures, and body aches.

**Table 2:** Themes and topics related to quality of life in patients with Gaucher disease

Theme	Topic	Main content of remarks	Example quote
Treatment status	Hearing impairment	Hearing loss	“Hearing loss is the biggest burden for me, but I can somehow hear things with hearing aids. I also think I’m causing a lot of trouble for everyone at work, but since I have told my work about my condition from the very beginning, I feel that I’m often getting a lot of help from others.” (Patient, type 3)
	Visual impairment	Squinted eyes, difficulty closing eyes	“It’s hard for him/her to close his/her eyes once they are open, or his/her eyes stay half open. So, we use eye drops frequently.” (Father, Type 2) “His/her eye movements (the way his/her eyes follow a moving object) are slow, so I think that is a symptom that I noticed. I think he/she isn’t good at it. For example, I noticed that his/her eye movements are slow when he/she is playing games.” (Mother, type 3)
	Difficulty swallowing	Sputum-sticking sensation while sleeping	“I think he/she doesn’t like having sputum caught in the throat, which he/she suffers from at night.” (Mother, type 3)
	Difficulty speaking	Difficulty talking	“I can talk like now, but I could not talk well before I started treatment. I felt like I was getting out of breath.” (Patient, type 3)
	Involuntary movement of extremities	Convulsive seizure, feeling that body and face are twitching	“His/her hands tremble, and he/she cannot walk properly. His/her movement is rough/sketchy, because he/she is not good at doing detailed work. Going to school has improved this a little, but he/she still has trouble with doing detailed things.” (Father, type 2)
	Epileptic seizures	Epileptic seizures; anxiety about onset or possibility of epileptic seizures at school/in work	“His/her symptoms are controlled by using a lot of medicines, but he/she starts to tremble when we try to reduce some of the medicines that he/she is using. That is his/her current situation. It was the worst when he/she was 2 years old; he/she experienced whole body rigidity and kept trembling (like a massive epileptic seizure) for 2 h. This happened about 6 times a day, almost every day for a year.” (Father, type 2) “When I used to have seizure attacks, I was in an ambulance twice a week, or even three times a week at worst.” (Patient, type 3)
	Body aches	Fatigue after treatment; body tilting; body stiffness; bone fractures occurring when only changing position	“His/her bones are very weak. When we took an x-ray, his/her bones looked like an eggshell. He/she has fractured his/her bones even from changing body positions. He/she has been fracturing his/her bones once every 3 months.” (Father, Type 2) “He/she sometimes tells me that his/her feet are painful. I don’t know if they are from the Gaucher disease, or if they are growing pains.” (Mother, type 3)
Patient burden	Anxiety about symptoms	Epileptic seizures	“The current treatment doesn’t seem to work on the neurological symptoms, but it seems to have worked on the hepatosplenomegaly. So, we are somewhat satisfied. It doesn’t work on the neurological symptoms, so it hasn’t helped him/her with the movement, or his/her swallowing difficulty; I’m not complaining but I’m worried about that.” (Mother, type 3)
	Difficulty with exercise and work	Anxiety about onset or possibility of epileptic seizures at school/in work	“Because I still experience few fractures, I can still work normally. But I always tell my workplace about my illness.” (Patient, type 3) “He/she was extremely poor at exercising. He/she wasn’t good at doing detailed tasks either… Every time he/she learned something new, there was something that he/she couldn’t do as much as others. He/she was eventually able to do it, but it took some time, like as if he/she was a slow starter.” (Mother, type 3)
	Anxiety about continuing treatment	Feel uneasy about how long treatment will be continued	“We don’t know when the clinical trial will end. We don’t know how many years it will take, and when this treatment will become available on the market.” (Patient, type 3)
	Anxiety about going out	I feel anxious about the risk of infectious diseases when going out, including going to the hospital	“We are worried about getting infectious diseases. Especially with infectious diseases, there is a risk of infection from going to the hospital, so we wish he/she could get treated at home without going to the hospital.” (Mother, type 3)
	Tiredness from hospital visit or treatment	Burden of traveling because the hospital that can provide enzyme replacement therapy is not in the neighborhood	“We need to take him/her to the hospital once every 2 weeks to receive enzyme therapy, and it is expensive because it costs about 300,000 yen. Considering that it is a rare illness, there are various complications. We have to take him/her to a specialized hospital, not a nearby hospital; we have to arrange a welfare vehicle to go to the hospital.” (Father, type 2) “I think it is pretty burdensome to get enzyme replacement therapy when he/she goes to an ordinary school. We decide on a day to go once every 2 weeks, so he/she cannot attend the class on that day.” (Mother, type 3)
Social support systems	Dissatisfaction about government service	As “Gaucher’s disease” is unrecognized as a disease name by government offices and public institutions, it is burdensome to explain	“When we take documents related to the system or subsidies to the government office, and we say “Gaucher disease”, we get told that there is no such thing because it is registered as lysosomal disease. In general, the person in charge of these welfare and intractable disease systems is replaced every 1–2 years, so this gets repeated again and again. So, it would be nice if there is a department that can handle all of these specialized diseases professionally if possible, and that can be said for any intractable diseases.” (Father, type 2)
	Lack of social support	Do not have information about support system	“When it comes to long-term care insurance service for elderly, or ≥ 40 years old, I think care managers are available to help out. There must be a lot of services available for these people. However, medical care for children is done by us a lot of the time, and the service that we can get depends on what information your attending physicians provide you with. So, I think it would be nice if someone could help with this.” (Father, type 2) “The thing I would like to get help with the most is school drop-off and pick-up from now on… Although it is nice to drop him/her off, and pick him/her up from school, he/she loves school, so I think he/she would find it exciting to go to school by himself/herself.” (Mother, type 2)
	Information exchange in patient association	Can exchange information or have a consultation with patients with the same disease type	“Even if we had the same disease type, patients experience different symptoms. There are people who are in a different situation from me. When I’m lost, I ask people who have similar symptoms as me. I ask what I should do and I feel a sense of security.” (Patient, type 3)

Themes and topics identified by qualitative analysis of patient interviews

Impaired hearing or vision was mentioned by one patient and three patients, respectively, and typical remarks included experiencing hearing loss, squinted eyes, or difficulty closing their eyes. Several patients reported difficulties with speaking or swallowing, which are often affected in nGD, especially type 2 [2, 17]. Seizures and involuntary movements, as well as anxiety about seizures, were also common concerns.

**Fig. 1:**
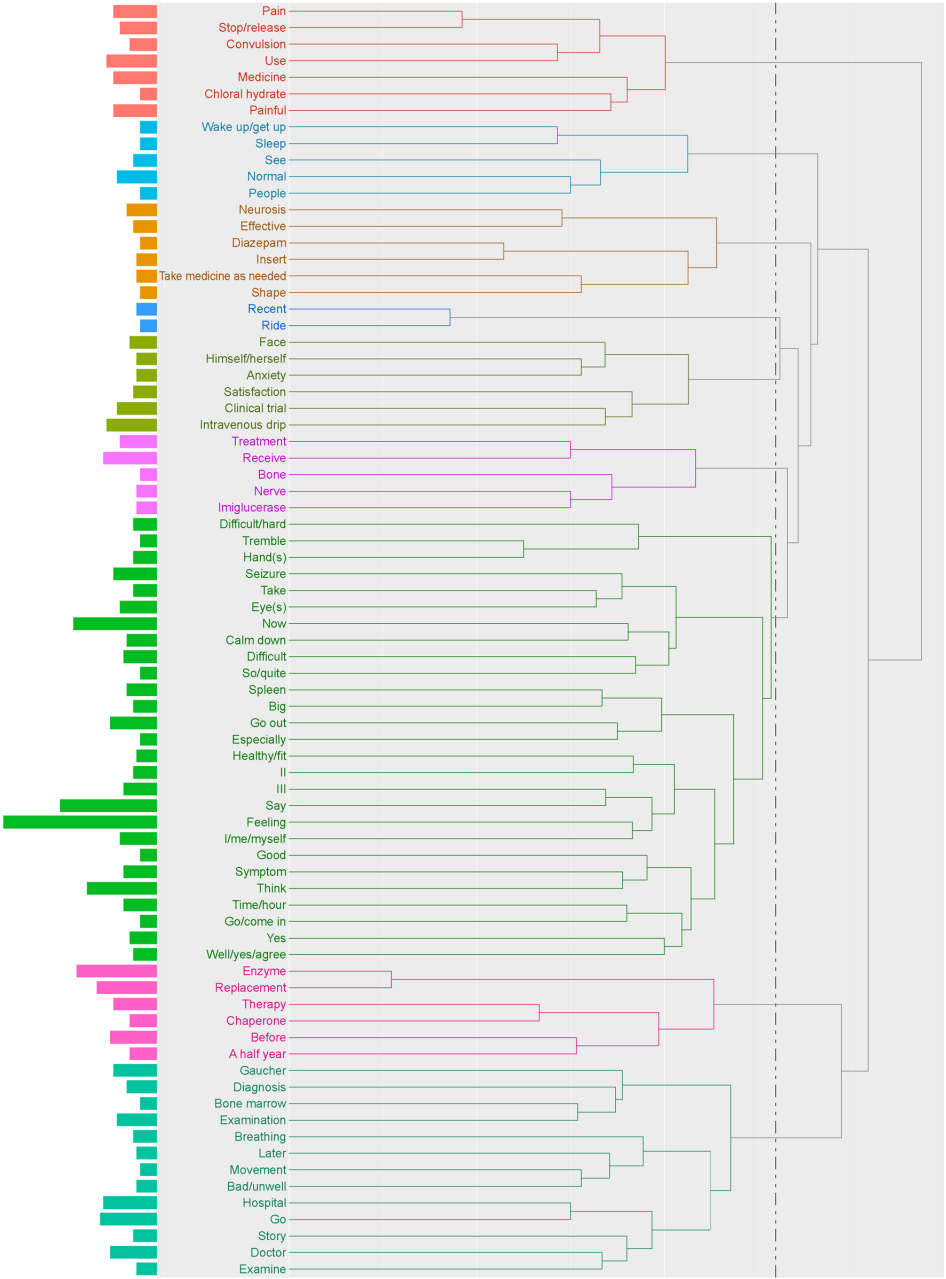
Hierarchical cluster analysis dendrogram of extracted words in the theme “treatment status”. The dendrogram indicates the result of cluster analysis based on the Ward method with Jaccard distance. The dotted line shows the cut-off for the level of cluster analysis. The length of the bars on the left side indicates the frequency of words

**Fig. 2:**
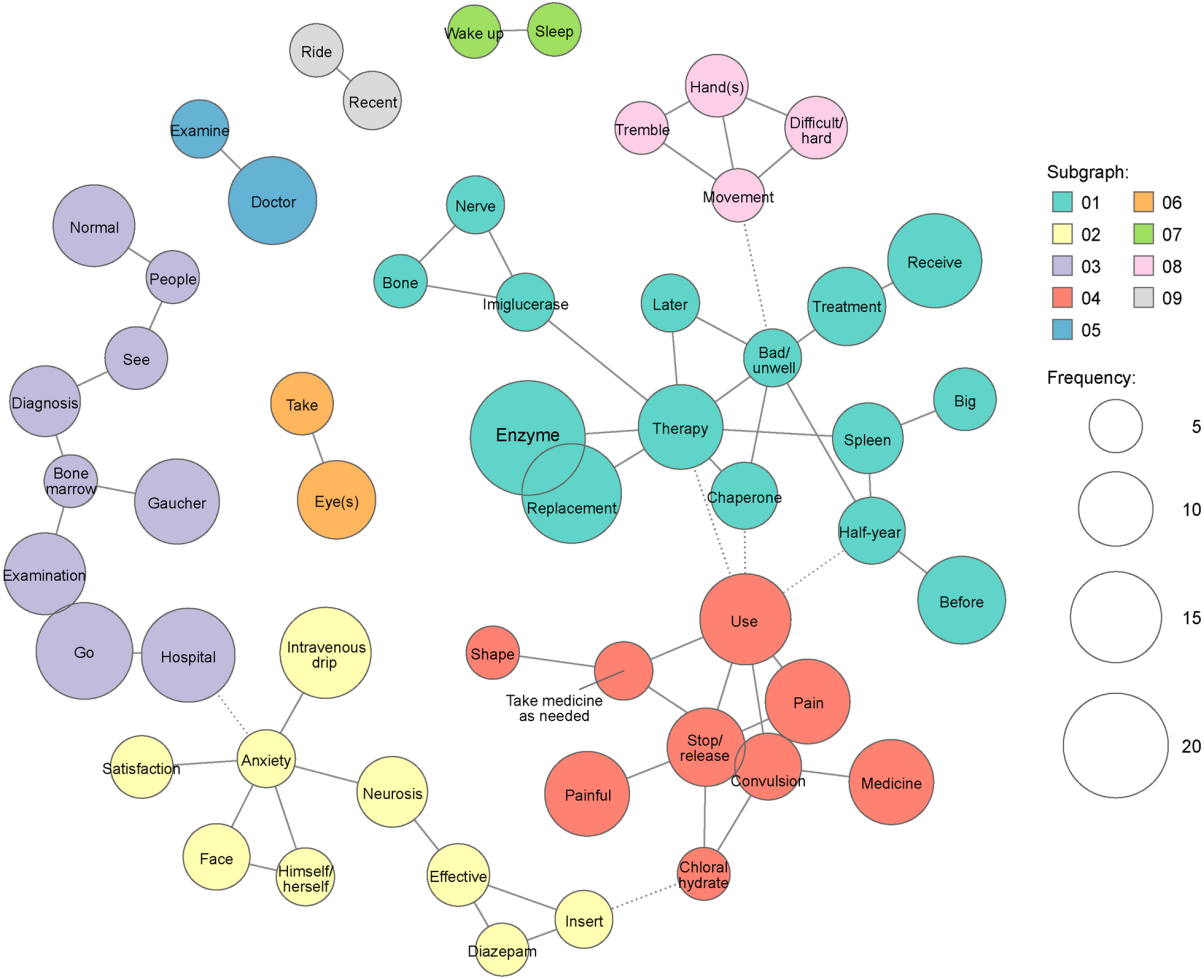
Co-occurrence network analysis of extracted words in the theme “treatment status”

Using hierarchical cluster analysis, nine clusters ranging from two to 27 words were identified for treatment status (Fig. 1). Among words that appeared ≥ 5 times, several words specific to neurological symptoms, such as “convulsions”, “hands”, “trembling”, “seizures”, and “spleen”, were mentioned, supporting the topics of “epileptic seizures” and “involuntary movement of extremities” identified in the qualitative analysis. In addition, medicines used for the treatment of seizures, such as “diazepam” and “chloral hydrate”, were mentioned. Words that appeared ≥ 5 times were assessed in a co-occurrence network to identify closely connected words related to treatments by medicine (Fig. 2). Specific word groups related to treatment were “enzyme”, “replacement”, “therapy”, “chaperone”, and “imiglucerase”. In addition, the term “medicines to be taken as needed” (one word in Japanese) was closely connected to “convulsions” and “pain”, suggesting that neurological symptoms are of concern to patients with nGD.

#### Patient burden

Five main topics emerged as common features of patient burden: anxiety about symptoms, difficulty with exercise and work, anxiety about continuing treatment, anxiety about going out, and tiredness from hospital visits or treatment. Several of the “patient burden” topics overlapped with “treatment status” topics, for example, anxiety about having an epileptic seizure. Indeed, four of the five topics were related to anxiety about various aspects of daily life, including worries about having seizures, particularly during school, work, or exercise, and the need for ongoing treatment. Patients also expressed concerns about the risk of infectious diseases when going out, even when visiting the hospital; this likely referred to COVID-19 as the interviews were conducted during the pandemic. Similar to the findings in the “treatment status” theme, fatigue after visiting the hospital or after treatment was common and was exacerbated when extended travel was required.

**Fig. 3:**
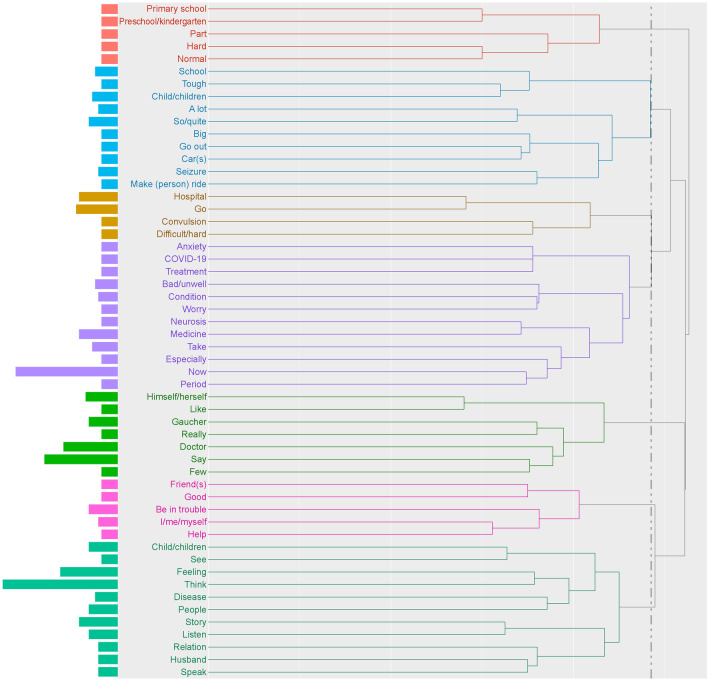
Hierarchical cluster analysis dendrogram of extracted words in the theme “patient burden”. The dendrogram indicates the result of cluster analysis based on the Ward method with Jaccard distance. The dotted line shows the cut-off for the level of cluster analysis. The length of the bars on the left side indicates the frequency of words

**Fig. 4:**
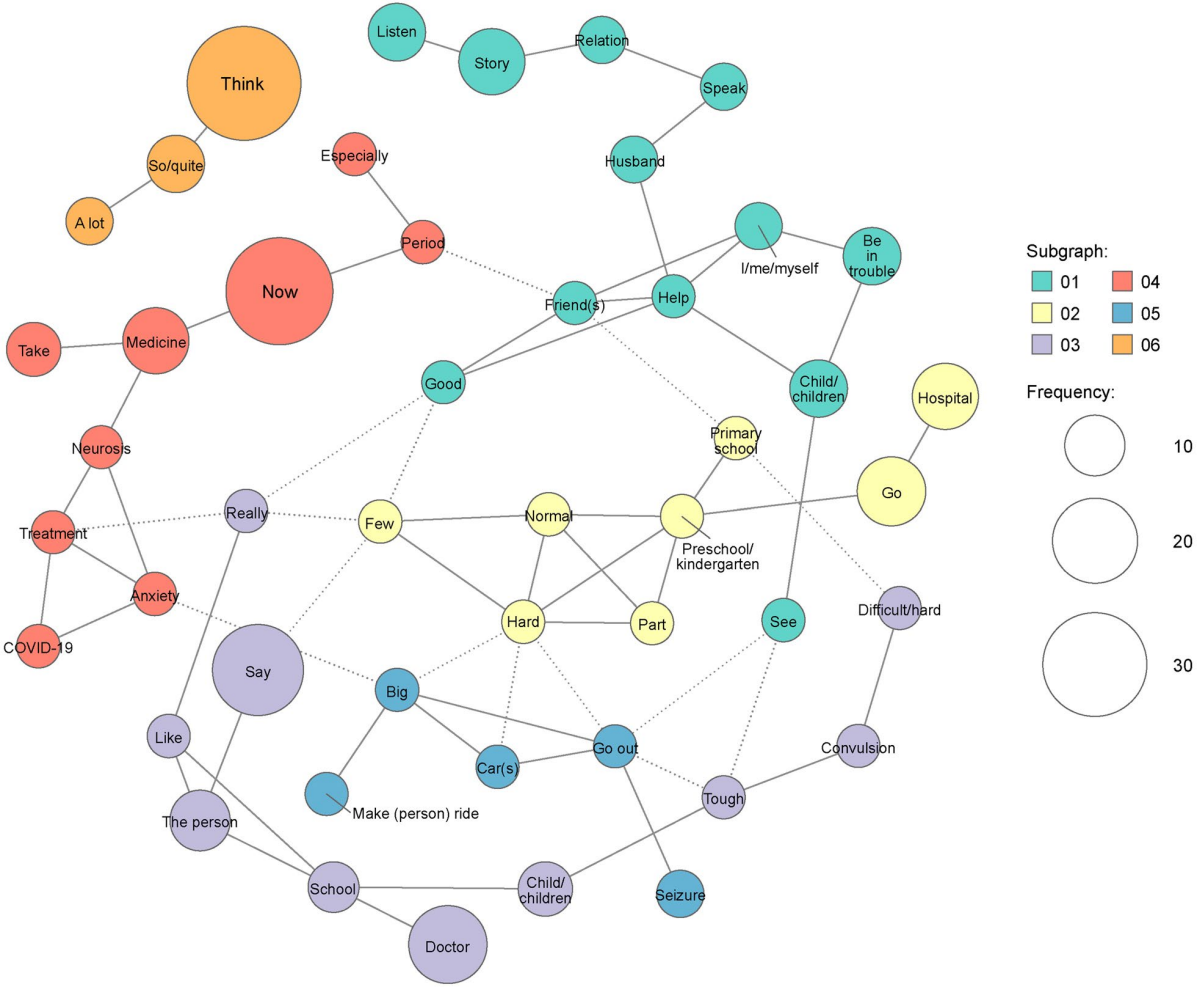
Co-occurrence network analysis of extracted words in the theme “patient burden”

Seven clusters ranging from four to 12 words were identified for patient burden (Fig. 3). Among the words mentioned ≥ 5 times, key clusters were identified for treatment burden and for the burden associated with schooling. Items related to treatment burden were hospital visit, from the words “hospital” and “difficult/hard” (one word in Japanese), and medication, from the words “take” and “medicine”. Items related to school burden were anxiety and difficulties at school from the words “school”, “tough”, “primary school”, “preschool/kindergarten”, and “hard”. For younger children with neurological symptoms, treatment burden and difficulties at school seemed to be inseparable. Words that appeared ≥ 5 times were included in the co-occurrence network (Fig. 4). Although some words were observed frequently, there were no words located at the center of the network. Each word was weakly connected to a wide range of other words.

#### Social support systems

Topics within the “social support systems” theme included dissatisfaction about government services, lack of social support, and information exchange in the patient association. Several patients indicated that the government services were inadequate and explaining GD to these services was burdensome. Although many patients said that information about social support systems was not available to them, some noted that they were able to exchange information with other patients through patient associations.

**Fig. 5:**
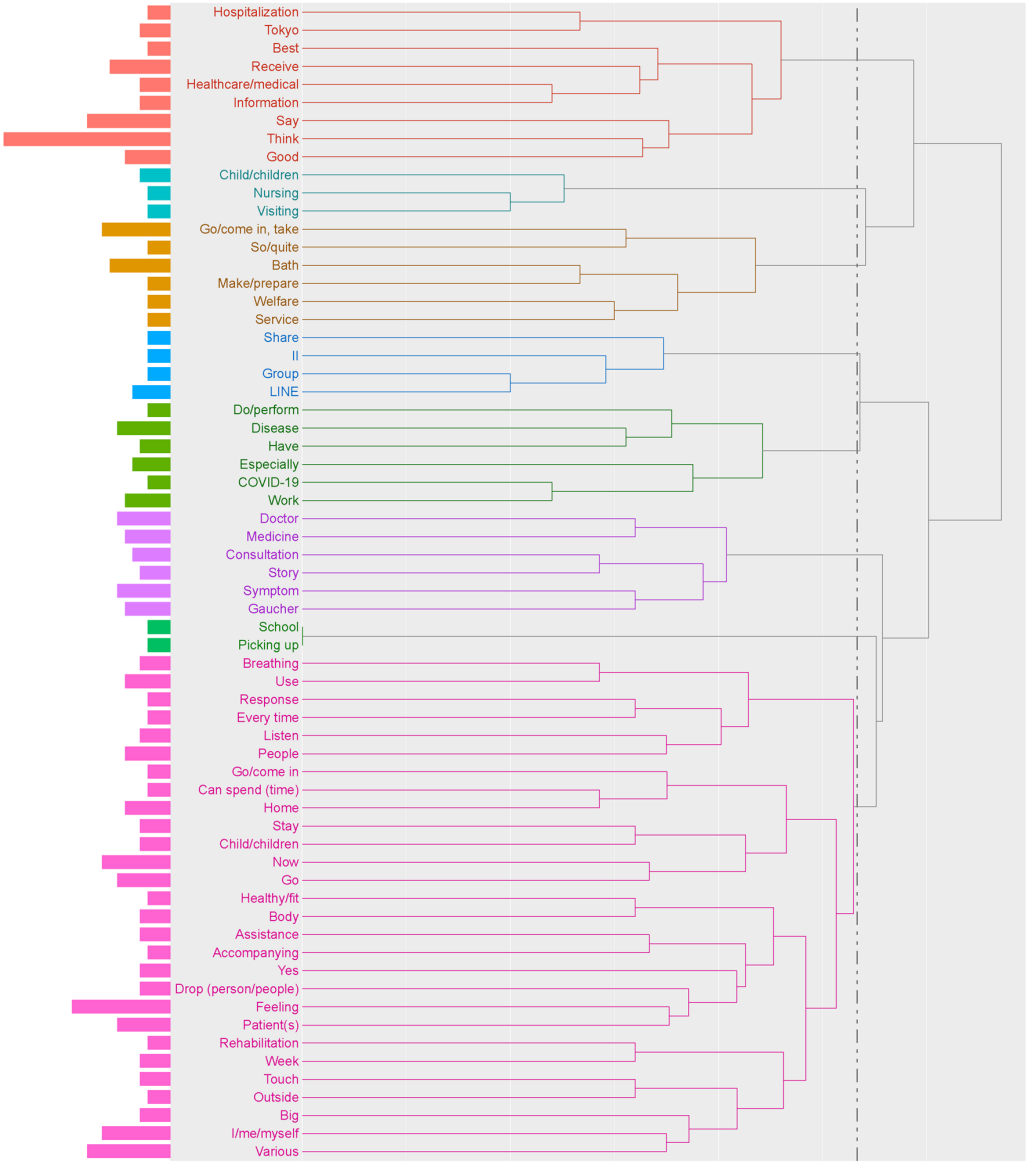
Hierarchical cluster analysis dendrogram of extracted words in the theme “social support systems”. The dendrogram indicates the result of cluster analysis based on the Ward method with Jaccard distance. The dotted line shows the cut-off for the level of cluster analysis. The length of the bars on the left side indicates the frequency of words. “LINE” is a messaging application used in Japan

**Fig. 6:**
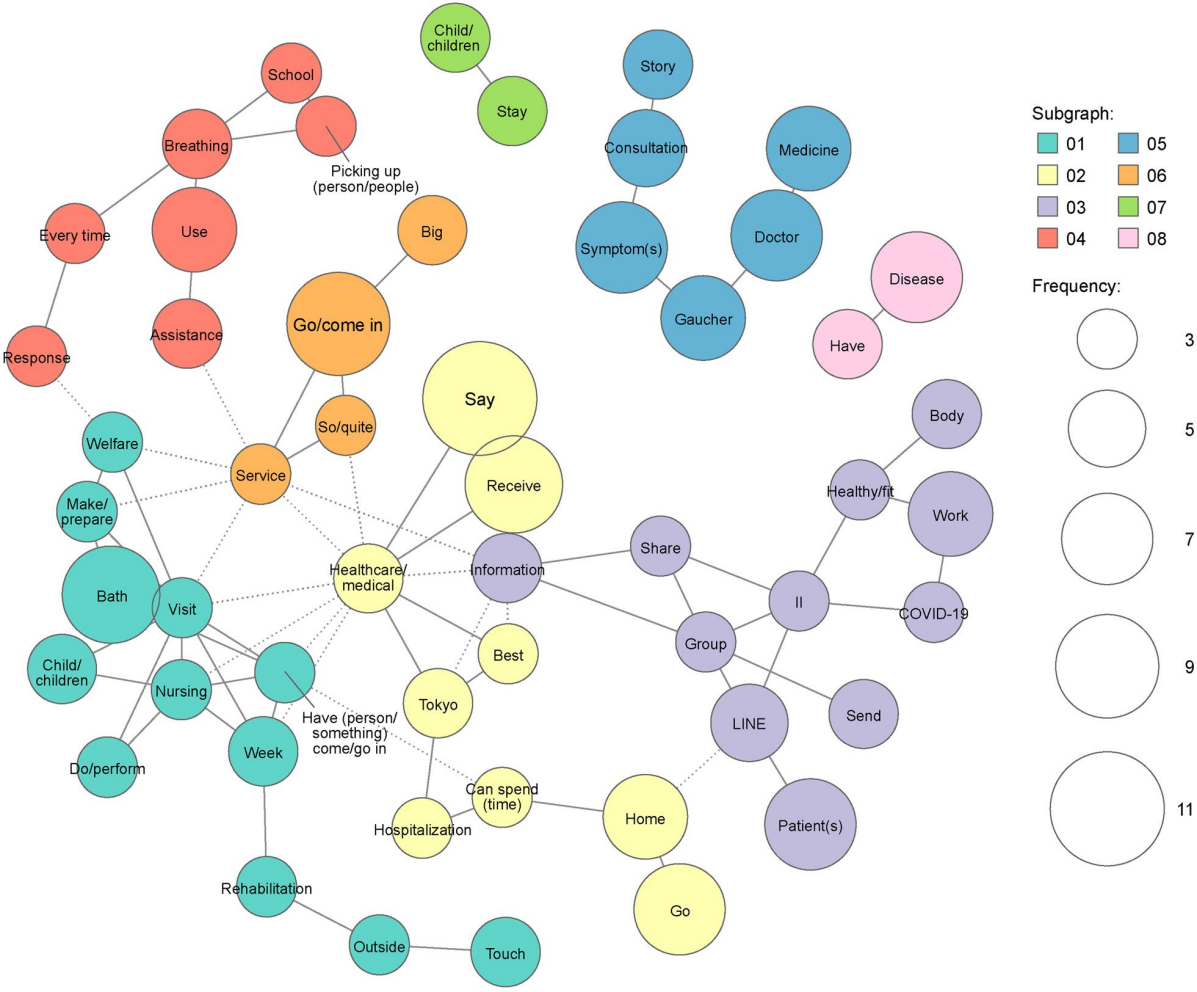
Co-occurrence network analysis of extracted words in the theme “social support systems”. “LINE” is a messaging application used in Japan

Eight clusters ranging from two to 28 words were identified for social support systems (Fig. 5). Among words that appeared ≥ 5 times, words specific to the types of services patients received, such as “visiting”, “nursing”, “bath”, and “rehabilitation”, were mentioned. In the co-occurrence network analysis (Fig. 6), words for services that patients received, such as “visiting”, “nursing”, and “bath”, were frequently mentioned together. In addition, words such as “doctor”, “symptom”, and “consultation” were frequently mentioned together, suggesting that patients and their caregivers considered the use of social support systems when consulting with their doctors. A cluster that included the words “LINE” (one of the most popular social networking services in Japan), “group”, “information”, and “share” suggested that information shared within the patient association was meaningful to patients and their caregivers and was utilized.

## Discussion

Using qualitative theme analysis of semi-structured interviews, coupled with hierarchical cluster and co-occurrence network analysis, this study has confirmed three main themes—treatment status, patient burden, and social support systems—that are important for patients with nGD and/or their caregivers. The results have allowed identification of key topics within each theme that will inform the development of a specific PROM; this can then be used to assess HRQOL and the effectiveness of medical and other interventions to improve the lives of patients, especially in Japan, where the proportion of patients with nGD is much higher than in other countries [6, 8].

The responses of the participants in this study were consistent with the actual treatment status of patients with nGD. For example, in the “treatment status” domain, enzyme replacement therapy was one of the main treatments mentioned for patients with types 2 and 3 GD. In addition to enzyme replacement therapy, other medicines, including chaperone therapy, were mentioned, which suggested that the treatments patients actually received were generally captured in the interview. This finding suggests that patients and caregivers who participated in the interview had an accurate understanding of the treatments available. However, despite treatment, the key topics we identified were all related to ongoing symptoms, suggesting that there is still a substantial unmet medical need. Enzyme replacement therapy can improve splenomegaly or hemoglobin concentration [18], but it has a minimal effect on neurological symptoms in nGD [17], including vision, hearing, swallowing, and seizures as reported by the patients in this study. The inclusion of questions in the PROM that relate to these symptoms will help quantify the current level of unmet need and evaluate the effect of newer treatments.

Findings from the analysis of the “patient burden” theme revealed that burden was associated not only with medicines, treatment, and having to attend outpatient clinics for treatment, as reported by others [9], but also with the impact of continuous infusion therapy on school attendance and performance. Patients with type 2 GD undergo intensive initial treatment, including enteral feeding, tracheostomy, and artificial ventilation, which places a high burden on caregivers [19]. Ongoing treatment with enzyme replacement therapy or other therapies, which requires patients to visit the clinic, is also associated with considerable burden [9, 19, 20]. Many of the key topics identified within this theme relate to anxiety, including anxieties about unexpected seizures, going out (particularly the risk of infection associated with going out), and the duration of continuing treatment. These anxieties are likely to have been heightened during the COVID-19 pandemic with the associated difficulties attending the clinic for regular treatment and uncertainty regarding the effect of COVID-19 infection on patients with GD [21]. Overall, our results emphasize the impact that nGD has on HRQOL beyond the physical symptoms.

In the “social support systems” theme, the results indicated that patients/caregivers considered the use of social support systems while consulting with their doctors or through peer exchange of useful information via the patient association(s) and social networking platforms (e.g. LINE). However, gaps between the current support system and patients’ expectations were also identified, particularly relating to a lack of information about what support systems are available and difficulties dealing with government services. Recently, base institutions for the treatment of incurable lysosomal diseases have been established, and it is expected that cooperation and information sharing among medical institutions regarding diagnosis and treatment of patients who live in the area might become more readily available. As Japan has a universal health insurance system, people can visit any doctor at any medical institution and receive medical services (treatment) without any restrictions; also, as Japan has adopted an “Act on Medical Care for Patients with Intractable Diseases” [22], patients with GD who receive enzyme replacement therapy have minimal costs for the treatment. Home-based enzyme replacement therapy, which patients with GD prefer [23], was approved by the Ministry of Health, Labour and Welfare in 2021 in Japan [24], but it is not yet widespread. In addition to support system development, other forms of patient support such as greater provision of structured care coordination, which can alleviate some of the burden on families of children with complex medical conditions, is urgently needed in Japan [25]. From the perspective of reducing family care burden, daily assistance with mobility or bathing and other day services are required for patients with advanced nGD [19]. Although some of these difficulties may be specific to Japan, it is likely that patients in other countries face similar obstacles to accessing the social support needed to make their lives easier.

This is the first stage in the development of a validated PROM for patients with nGD and their caregivers in Japan. The results of this analysis have confirmed the need to include additional questions in the current PROM questionnaires that address the impact of neurological symptoms, the burden caused by these symptoms, and patient needs related to social support systems. The current questionnaire for patients with type 1 GD has no questions related to current symptoms, including neurological symptoms, caused by GD [13, 14]. As patients with types 2 and 3 GD are generally younger than those with type 1, this study has also provided insights into the burden associated with GD that may be specific to the daily lives of younger patients. Although the study was based on a small number of participants, the patients were recruited from a patient association and therefore were considered to represent the real-life opinions and concerns of patients with GD in Japan. No qualitative research has been done for patients with types 2 and 3 GD, whereas there are some reports for patients with type 1 GD [9, 10, 20].

This study does have some limitations. Although only eight patients/caregivers were included in the interview, this number is considered sufficient for content validity based on the COSMIN checklist [16]. The participants were limited to patients registered with the patient association and those who consented to participate in web or phone interviews during the specified period. Conducting the interviews by web or phone limited the ability of the interviewer to observe non-verbal cues. In this study, only patients currently being treated by a physician were included. For all patients < 16 years old, interviews were conducted with a caregiver and did not take into account potential discordance between the patient’s and caregiver’s perceptions of patient burden. Although the domains identified in the analysis are likely to be relevant to patients with nGD worldwide, the analysis was specific to the Japanese language and Japanese clinical setting. Because the interviewer was a nurse, the patient may have been biased toward answering items related to medical care and treatment rather than general daily life. The interviewer had previously been involved in the care of one patient with type 3 GD but was not currently involved. Although it was possible for the previous involvement to influence the content of the interview, it is unlikely to have negatively affected the purpose of this study, i.e. obtaining responses to questions for the development of a patient-reported outcome measure. Finally, because patients were aware that the study was sponsored by a pharmaceutical company (as disclosed in the recruitment pamphlet), it is possible that they may have suppressed negative statements about treatments.

## Conclusions

In conclusion, the semi-structured interview format was able to provide an objective analysis of the burden of nGD. The themes and topics identified in this analysis were specific to patients with nGD and will be used to inform the development of a HRQOL questionnaire for these patients. Ultimately, we plan to combine the nGD questionnaire with the recently published type 1 questionnaire [13] to produce a single PROM that can be used for all patients with GD in Japan and elsewhere.

## Methods

### Study design

This observational study consisted of three stages: (1) qualitative interviews with patients with nGD, (2) pre-testing of the questionnaire in patients and caregivers, and (3) field testing of the questionnaire in patients and caregivers. The purpose of the qualitative interview was to determine which domains in the current GD PROMs were relevant to the target study population to inform the development of a Japan-specific HRQOL questionnaire for GD. For the first stage, in-depth 1:1 patient interviews were conducted using a qualitative, semi-structured interview method to gain insights from participants about disease burden and how they currently share their QOL information with physicians. Data from qualitative interviews were analyzed using a thematic analysis method. The interviews were conducted in Japan from February 1, 2021 to March 8, 2021.

The study is registered at the UMIN Clinical Trials Registry (https://www.umin.ac.jp/ctr/index.htm [UMIN000042872]). The protocol and informed consent form were approved on January 13, 2021 (No. 20342) by Osaka University Clinical Research Review Committee. The study was conducted in accordance with the ethical principles that have their origin in the Declaration of Helsinki, the Guidelines for Good Pharmacoepidemiology Practices, and all applicable laws and regulations. All data collected during the study were de-identified and anonymized.

### Study population

Male or female patients were eligible for inclusion if they had a confirmed diagnosis of type 2 or 3 GD, were diagnosed by a physician, and had been treated for GD. For patients < 16 years old, a caregiver (≥ 20 years old) or family member with a good understanding of the patient participated on behalf of the patient. Any participant with cognitive disabilities, who lacked fluency in Japanese, or who was judged by the investigator to be unsuitable for any other reason was excluded. Patients or their legally acceptable representatives could only participate in the study once. All participants were required to be capable of understanding our explanation of the purpose of this study and of complying with protocol requirements. Patients or their legally acceptable representatives, if applicable, were required to provide written informed consent to participate in the study and agree to the privacy authorization before commencing the study.

### Qualitative interviews

Participants were recruited by referral from a patient association in Japan (Association of Gaucher Disease Patients in Japan). Given the physical difficulties some patients had in accessing the interview venue and the risk of infection during the ongoing global COVID-19 pandemic, most study activities were conducted via online web-based platforms. Invitations were sent by mail, and written paper-based informed consent was obtained. Screening questions were asked after written consent and before scheduling 1:1 in-depth interviews to make sure the participants met the eligibility criteria. On the day of the in-depth interviews, informed consent was reconfirmed and, if needed, interviewers provided answers to consent-related questions from the participants.

Interviews were conducted in Japanese by one of the authors, Y. Koto, who has been involved as a nurse in enzyme replacement therapy for patients with type 3 GD. The interviewer was not involved in the care of the patients who were interviewed, except for one patient with type 3 GD with whom the interviewer had been involved previously. Independent, qualified interviewers (IQVIA Solutions Japan K.K.) provided support for the interviews under the guidance of authors A. Narita and N. Sakai, who are GD specialists with experience in the treatment of patients with GD. The interviewers led and facilitated the interviews, and only information related to the study objective was collected. The interviews were conducted via an online meeting system (WebEX) or by telephone using a pre-prepared interview guide (see Additional file 1).

The interview was developed by the authors, who are GD specialists and/or experts in qualitative analysis, and was based on existing PROMs for type 1 GD [13, 14]. The interview guide focused on three main themes, which were confirmed by analysis of interview transcripts. The interview took approximately 30–40 min for each participant. The interview was audio recorded with participant approval and transcribed in its entirety in Japanese. Transcribers omitted any data from the transcripts that may have led to identification of participants. Each participant who completed an interview received a gift card for 2,000 yen.

### Statistical analysis

A total of 10 patients with types 2 and 3 GD (five patients each) were to be interviewed. As the purpose of this study was to elicit relevant evidence from qualitative interviews, a sample size of convenience was applied. Given that there are approximately 200 patients with GD of all types in Japan [6], 10 unique individual interviews were considered a large enough pool to investigate relevant opinions.

The transcripts of all interviews were manually confirmed. We set the interview framework to obtain participants’ experience as extensively as possible. When the transcript texts were classified into the three main themes proposed in advance, we confirmed that the themes were adequate and that no other themes were necessary. For each theme, a coding list was created using text-mining software for Japanese language data (KH coder version 3.Beta.03, updated September 7, 2021, Higuchi Koichi, Japan) [26, 27]. Coding was based on attribute information and the presence or absence of words corresponding to specified coding rules. Coding rules were specified by manually extracting words after confirmation among analysts.

Hierarchical cluster analysis and co-occurrence network analysis were performed to extract frequently occurring and co-occurring words; the resulting associations between words within sentences were visually represented on a co-occurrence network map. In the hierarchical clusters, words that appeared ≥ 5 times (the default setting in KH coder) were clustered by the Ward method, and the Jaccard distances between clusters were calculated. In the co-occurrence network analysis, for words that appeared ≥ 5 times and their word combinations, the co-occurrence relation was calculated by the Jaccard index, and up to 60 co-occurrence relationships were drawn as lines from the relationship with the largest Jaccard index.

## Supplementary Information

**Additional file 1**. Interview guide for patients—English version (translation).

## Data Availability

The data generated and/or analyzed during the current study are available from the corresponding author on reasonable request.

## Abbreviations

GD: Gaucher disease
HRQOL: Health-related quality of life
nGD: Neuronopathic GD
PROM: Patient-reported outcome measure
QOL: Quality of life
