# Correction: Qualitative analysis of patient interviews on the burden of neuronopathic Gaucher disease in Japan

**DOI:** 10.1186/s13023-023-02659-9

**Published:** 2023-03-24

**Authors:** Yuta Koto, Aya Narita, Shinichi Noto, Midori Ono, Anna Lissa Hamada, Norio Sakai

**Affiliations:** 1 Child Healthcare and Genetic Science Laboratory, Division of Health Sciences, Osaka University Graduate School of Medicine, 1-7 Yamadaoka, Suita, Osaka 565-0871 Japan; 2 Division of Child Neurology, Institute of Neurological Science, Faculty of Medicine, Tottori University, 36-1 Nishi-cho, Yonago, Tottori 683-8504 Japan; 3 Department of Rehabilitation, Niigata University of Health and Welfare, 1398 Shimami-cho, Kita-ku, Niigata, Niigata 950-3198 Japan; 4 Japan Medical Office, Takeda Pharmaceutical Company Limited, 1-1 Nihonbashi-Honcho 2-chome Chuo-ku, Tokyo, 103-8668 Japan

**Correction: Orphanet Journal of Rare Diseases (2022) 17:280** https://doi.org/10.1186/s13023-022-02429-z

Following publication of the original article [1], we have been notified that there was typo mistake in Table 1. It should be mentioned as—walk without assistance in the daily activity.
