# Supplementary material for: Qualitative analysis of patient interviews on the burden of neuronopathic Gaucher disease in Japan
Source: Orphanet J Rare Dis. 2022 Jul 19;17:280. doi: 10.1186/s13023-022-02429-z (PMC9295096; doi:10.1186/s13023-022-02429-z)
Supplement: Supplementary file 1 — Additional file 1. Interview guide for patients—English version (translation). [file 13023_2022_2429_MOESM1_ESM.docx]

Additional file 1.

**Interview Guide for Patients – English Version (translation)**

| **Time** | **Item** |
| --- | --- |
| 2 min | 1. **Interview description**  - Thank you for attending the interview today. - (Only if the consent form has not been received)  I mailed the consent form for the interview the other day; did you receive it?   Did you send it back?   - - - (If returned) It seems that it has not arrived as of yesterday. We need to confirm that you have given your consent before proceeding with the interview, so please allow me to read the consent form aloud now.   (Consent Form Reading)  Do you consent, as per the details I have just read to you?   - - - (If not returned) Please allow me to read the consent form aloud now, as it is necessary to confirm that consent has been obtained before proceeding with the interview.   (Consent Form Reading)  Do you consent, as per the details I have just read to you?  You will also be required to provide written informed consent as a record of your consent. Could you please return the consent form with today's date and your signature at your earliest convenience?   - During this interview, you will be asked mainly about the burden of Gaucher disease and the symptoms that cause this burden. Using the results of the interview, we will create a scale to measure the patient’s perceived burden, and in the future, we aim to provide more patient-oriented medical services. - There are no right or wrong answers. I would appreciate it if you could share your thoughts in response to my questions. - The interview takes approximately 30–40 minutes. - Your answers are valuable to us, but it is difficult to note down everything, so we will be recording the interview. Please be assured that recordings will only be used for the purpose of analysis and will not be used for any other purposes. - If you mention side effects, etc. related to a specific medicine during the interview, we will report the details to Takeda Pharmaceutical Company Limited. Thank you for your understanding. - You are not obliged to answer any questions you do not want to answer during the interview. You are welcome to stop the interview at any time. Please do not hesitate to inform us of any issues, including if you feel unwell. - That is everything I need to explain about the interview. Do you have any questions? |
| 3 min | 1. **Personal information**   **Presence or absence of companion**   - I would now like to start the interview. Are you alone, or are you accompanied by someone?   - - (If accompanied) Who are you with?   **Personal information**   - The disease burden varies depending on the patient’s age, environment, symptoms, etc., so allow me to first ask you some questions about yourself in order to understand the burden. - Which prefecture do you live in? - What is your age? - (If you are under 18 years old) Do you usually go to school?   (For those aged 18–22 years) Do you usually go to school or do you work? (For those 23 and older) Do you work?   - (If working) What kind of work do you do? - Which family members do you live with? - Are any of your family members living with you able to support you in your daily life or treatment for Gaucher disease?   - - Who supports you?     - In what way? |
| 5 min | 1. **Current treatment status**   **Treatment history**   - I am now going to ask you about Gaucher disease. Do you know when you were first diagnosed with Gaucher disease? - When did the treatment start? - There are several types of Gaucher disease (e.g., type 2 and type 3). Which type do you have?   **Subjective symptoms**   - How is your current physical condition? Are there any problems? Do you have any symptoms?   - - Check for symptoms offered spontaneously: check the extent of the symptoms and the frequency with which they occur.   If the patient has few spontaneous comments about symptoms, confirm the following as well. If symptoms are present, also check for specific symptoms:   - Do you feel that your stomach becomes full quickly or that you lose your appetite? - Do you tire easily or feel sleepy easily? - Do you have any pain? For example, is there numbness in your hands and feet or do you have bone pain? - Do you have any sensation of swelling in your stomach? - What about body movement? Do you find that you cannot move your body as you would expect or that your body tenses up on its own? - Do you have any concerns relating to breathing?   **Current treatment of symptoms**   - What kind of treatment (for the symptoms you mentioned) are you receiving?   - - (If not mentioned) Is it oral or intravenous?     - Do you know the name of the medicine (if applicable)?^a^ - How satisfied are you with your current treatment?   - - What are you satisfied (not satisfied) with? |
| 15 min | 1. **Patient burden**   **Patient burden**   - What are some of the burdens of having Gaucher disease in your daily life?   *If the patient seems confused about how to answer the question, ask him/her: “Can you talk about what is troubling you, what you are not able to do as you would like, or what you are worried about because of Gaucher disease?”   - - - Confirm the burden that was mentioned by the patient spontaneously: When do you feel that way?   If the patient has few spontaneous comments, confirm the following as well.  Be specific about each:   - We just talked about the symptoms, but are there any symptoms that you felt were painful?   - - What is the impact of the symptoms on your daily life? - Do you feel anxious or depressed because of Gaucher disease? - Are there any limitations on you due to having Gaucher disease? - Are there times when you hesitate or give up on what you want to do? - Does Gaucher disease affect your relationship with others? - Are there any financial burdens? - Do you feel that regular hospital visits and treatment are a burden?   **Risk of complications**  If concerns about complications are not mentioned, ask:   - Has your attending physician mentioned anything about future medical conditions? Do you have any other concerns in addition to what your attending physician has told you?   - - How do you feel about that?   **People that the patient can talk to**   - Is there anyone you can talk to when you need help with the burden you mentioned earlier?   - - Who is that? |
| 5 min | 1. **Support systems, etc.**   **Welfare services**   - Are you currently receiving any welfare services?  Add the following description depending on the response: Welfare services include care visits, provision of welfare equipment, and living assistance provided by the government, etc.   - - What services are you receiving?     - How do those services help you in your life?   **Assistance needs**   - As a patient living with Gaucher disease, do you need any additional assistance from local government, healthcare professionals, or the people around you?   - - What kind of support would be helpful?     - Who would you like to receive support from and what kind of support?   **(If time permits) Patient association**   - I believe that you have joined a patient association.   - - How do you engage with other members?     - What do you like about being part of the patient association? |

^a^ All medicine brand names were converted to generic medicine names during data processing
